# Synthetic Reconstruction and Characterization of Endogenous Bacteriophage Genomes from *Desulfovibrio vulgaris*

**DOI:** 10.3390/v18101159

**Published:** 2026-10-19

**Authors:** Butaek Lim, Tekle Fida, Karen Crippen, Rebekah Wilson, Seung-Wuk Lee

**Affiliations:** 1 Department of Bioengineering, University of California, Berkeley, CA 94720, USA; 2 Biological Systems and Engineering Division, Lawrence Berkeley National Laboratory, 1 Cyclotron Rd, Berkeley, CA 94720, USA; 3 Gas Technology Institute, 1700 South Mount Prospect Rd, Des Plaines, IL 60018, USA; 4 US Army Corps of Engineers, Champaign, IL 61820, USA

**Keywords:** bacteriophage, prophage induction, *Desulfovibrio vulgaris*, sulfate-reducing bacteria, microbiologically influenced corrosion, synthetic genome assembly, PHASTEST, phage genome engineering

## Abstract

Microbiologically influenced corrosion (MIC) caused by sulfate-reducing bacteria (SRB) is a major challenge in industrial infrastructure, yet the limited availability of well-characterized bacteriophages has hindered the development of phage-based strategies for controlling these organisms. Here, we present an integrated genome-guided workflow for the discovery, validation, and synthetic reconstruction of endogenous prophages from the model sulfate-reducing bacterium *Desulfovibrio vulgaris* Hildenborough. Bioinformatic analysis using PHASTEST identified seven prophage-like regions distributed throughout the bacterial chromosome, including five classified as intact prophages. Ultraviolet (UV)-mediated induction followed by whole-genome sequencing of purified phage DNA experimentally validated four inducible prophage loci (Regions 1, 5, 6 and 7) detected in all three biological replicates, together with a fifth locus (Region 2) detected less consistently, demonstrating strong agreement between computational prediction and biological activity. Comparative analysis further showed that prophage functionality was not always correlated with computational completeness, as one computationally “questionable” region exhibited robust induction whereas another “intact” region remained inactive under the conditions tested. Based on these results, one highly inducible prophage was selected for synthetic engineering. Three progressively truncated synthetic prophage genomes (approximately 36, 33, and 29 kb) were assembled using long-range PCR and scarless isothermal DNA assembly and verified by Nanopore sequencing, which confirmed accurate reconstruction without detectable rearrangements. Following electroporation into *D. vulgaris* and UV induction, the engineered genomes produced reproducible localized turbid phenotypes rather than classical lytic plaques, indicating that the synthetic constructs remained biologically active but did not undergo efficient lytic propagation under the experimental conditions examined. Quantitative PCR analysis of representative structural and lytic genes in wild-type cells revealed coordinated transcriptional activation by 21 h after UV induction, defining a practical harvest time for subsequent characterization. Collectively, this work establishes an integrated computational and synthetic biology framework for engineering endogenous prophages from anaerobic SRB. The approach provides a foundation for investigating temperate phage biology in *D. vulgaris* and represents an important step toward the future development of engineered phage platforms for MIC control.

## 1. Introduction

Microbiologically influenced corrosion (MIC), also referred to as biocorrosion, is a major cause of infrastructure deterioration in the oil and gas, maritime, wastewater, and energy industries, resulting in substantial economic losses and safety risks worldwide [1,2,3]. Unlike purely electrochemical corrosion, MIC arises from the metabolic activities of microorganisms that colonize metal surfaces and form complex biofilms. Among these microorganisms, sulfate-reducing bacteria (SRB) are considered one of the critical biological drivers of anaerobic corrosion [2,3,4,5]. In particular, *Desulfovibrio vulgaris*, one of the best-characterized SRB species, accelerates corrosion through sulfate respiration, producing hydrogen sulfide while facilitating extracellular electron transfer from metallic surfaces. These processes promote localized pitting corrosion, structural degradation, and premature failure of industrial equipment [3,4,5].

Current strategies for controlling SRB-associated corrosion rely largely on the repeated application of chemical biocides, including glutaraldehyde, quaternary ammonium compounds, and oxidizing agents [6,7]. Although these treatments can reduce microbial populations, their long-term effectiveness is often limited by incomplete penetration into mature biofilms, environmental toxicity, high operational costs, and the emergence of biocide-tolerant microbial communities [3,7]. Consequently, there is growing interest in developing alternative antimicrobial strategies that selectively target corrosion-causing microorganisms while minimizing impacts on surrounding microbial ecosystems.

Bacteriophages (phages), the natural viruses of bacteria, represent an attractive biological alternative because of their exceptional host specificity and self-amplifying mode of action [8,9,10,11]. Lytic phages infect susceptible bacteria, replicate intracellularly, and release progeny virions through host cell lysis. In addition, many phages encode depolymerases and other enzymes capable of degrading extracellular polymeric substances, potentially improving penetration into bacterial biofilms [12,13]. These properties have generated increasing interest in the application of phages for biofilm control, environmental biotechnology, and antimicrobial therapy [11,14].

Despite this promise, relatively few lytic phages have been isolated and characterized for SRB, particularly anaerobic species such as *D. vulgaris*. The limited availability of naturally occurring virulent phages has become a significant obstacle to developing phage-based approaches for controlling MIC [15,16,17]. In contrast, many bacterial genomes harbor integrated temperate bacteriophages (prophages) that remain dormant until induced by environmental stresses, such as DNA damage [18,19,20]. These endogenous prophages represent an alternative source of phage genomes that can potentially be exploited for synthetic engineering to generate targeted MIC-controlling agents.

Recent advances in genome mining and synthetic biology have substantially expanded the ability to identify and manipulate endogenous prophages. Computational platforms such as PHAge Search Tool with Enhanced Sequence Translation (PHASTEST) enable systematic identification and annotation of prophage regions based on sequence homology, viral gene organization, and structural signatures [21,22]. When combined with experimental induction and genome engineering technologies, these tools provide opportunities to reconstruct endogenous prophages and evaluate their biological activity without relying exclusively on environmental phage isolation [23,24,25]. Furthermore, synthetic genome assembly enables targeted modification of prophage genomes, providing a framework for investigating the genetic determinants of phage induction, replication, and host interactions [26,27,28,29,30,31].

In this study, we developed an integrated computational and experimental pipeline for the identification, validation, and synthetic reconstruction of endogenous prophages from *Desulfovibrio vulgaris* Hildenborough. First, prophage regions were identified using PHASTEST and experimentally validated through ultraviolet (UV)-mediated induction followed by whole-genome sequencing of purified phage DNA. Comparative analysis between computational predictions and experimentally recovered viral genomes identified the major inducible prophage loci within the chromosome. Based on these results, one highly inducible prophage was selected for synthetic reconstruction, and a series of progressively truncated genomes was assembled using long-range PCR and scarless isothermal DNA assembly. The engineered genomes were introduced into *D. vulgaris*, and their biological activity was evaluated through phenotypic assays and quantitative PCR analysis of prophage gene expression.

Together, this work establishes a genome-guided framework for discovering and engineering endogenous prophages from SRB. Rather than relying on environmental isolation of naturally occurring phages, our approach combines bioinformatic prediction, experimental validation, synthetic genome reconstruction, and functional characterization into a unified workflow. This strategy provides a foundation for future development of engineered phage platforms for studying phage biology in anaerobic bacteria and may ultimately contribute to phage-based approaches for controlling MIC.

## 2. Materials and Methods

### 2.1. Bacterial Strain, Culture Conditions, and Cell Line Authentication

A verified, contamination-free isolate of *Desulfovibrio vulgaris* Hildenborough (NCBI accession NC_002937.3) was obtained from the American Type Culture Collection (ATCC, Manassas, VA, USA; strain ATCC #29579) and used as the source strain for all experiments. Cells were retrieved from freeze-dried stock inside an anaerobic glovebox maintained under a 10% CO_2_/90% N_2_ atmosphere. Two growth media were used for distinct purposes. Postgate medium B (PGM) was used for revival from freeze-dried stock and for routine subculture. Coleville synthetic brine medium K (CSBK) was used for all experimental cultures, including UV induction, electroporation recovery, and the phenotypic and transcriptional assay reported here. Both were prepared as described previously [32] and purged with CO_2_/N_2_ for 30 min prior to inoculation. Freeze-dried cell pellets (10–15 mg) were resuspended in 200 µL of purged medium inside the glovebox, transferred into 20 mL of purged CSBK or PGM medium via syringe, purged for an additional 10 min to remove residual molecular oxygen, and incubated at 30 °C for 4–6 d until visible growth was observed. Cultures were maintained anaerobically thereafter and subcultured every 4–6 d.

Hydrogen sulfide production was monitored at day 3 of each passage using a colorimetric methylene blue assay to confirm active sulfate reduction. Cell line identity and purity were confirmed at each passage by extracting genomic DNA, amplifying the 16S rRNA gene by PCR using universal primers 8F (5′-AGAGTTTGATCCTGGCTCAG-3′) and 1492R (5′-GGTTACCTTGTTACGACTT-3′), and Sanger sequencing the resulting 1.4 kb amplicons. Sequences were compared against the NCBI nucleotide database using BLASTn and the *D. vulgaris* 16S rRNA reference sequence (accession NR_074446.1) to confirm strain identity and exclude contaminating organisms across all passages used in this study.

### 2.2. Bioinformatic Prediction of Prophage Regions

The complete *D. vulgaris* Hildenborough chromosome sequence (NC_002937.3) was submitted in FASTA format to PHAge Search Tool with Enhanced Sequence Translation (PHASTEST: https://phastest.ca, accessed on 9 August 2024) [21,22]. The native megaplasmid pDV1 (NC_005863.1) was not analyzed; all prophage counts reported here therefore refer to the chromosome. Coding sequences, tRNA genes, and other genomic features were annotated using GLIMMER [33], tRNAscan-SE [34], and ARAGORN [35], and putative viral open reading frames were identified using BLAST (BLAST 2.17.0)-based similarity searches against the PHASTEST curated bacteriophage protein database. Candidate regions were scored and classified as intact, questionable, or incomplete based on the number of coding sequences, the density of phage-associated keywords (e.g., capsid, integrase, tail, portal, terminase, lysin), region length (>30 kb), total predicted proteins (≥40), and the proportion of phage-related and hypothetical proteins (>70%), consistent with the standard PHASTEST scoring algorithm. Chromosome-wide GC content and GC skew [(C − G)/(C + G)] were computed using a 1 kb sliding window across the full chromosome and rendered alongside circular and linear genome maps of predicted prophage boundaries.

### 2.3. UV-Mediated Prophage Induction and Whole-Genome Sequencing

Four independent 0.2 L *D. vulgaris* cultures were grown anaerobically to late-log/early-stationary phase (OD600 = 1.0–1.2) and UV-irradiated using a handheld 254 nm UV lamp (UVP, Upland, CA, USA, UVGL-25) positioned 20 cm above the culture surface for 1 min, with the vessel opened and stirred to induce the bacterial SOS response and trigger prophage excision. The manufacturer’s rated 254 nm irradiance for this lamp is 0.72 mW/cm^2^ at 7.6 cm; scaling to the 20 cm working distance by the inverse-square relationship gives an estimated irradiance at the culture surface of approximately 0.10 mW/cm^2^, corresponding to an estimated surface fluence of approximately 6.2 mJ/cm^2^ (62 J/m^2^). Irradiance was not measured radiometrically and the value is therefore reported as an estimate. The same lamp, distance and exposure were used for all induction experiments. Following irradiation, cultures were incubated anaerobically for 24 h to allow phage particle production. Cell debris was removed by centrifugation followed by filtration through a 0.22 µm syringe filter. Residual host DNA and RNA in the resulting phage-containing supernatant were degraded by treatment with DNase I (NEB, Ipswich, MA, USA, 5 units/mL) and RNase A (Sigma Aldrich, St. Louis, MO, USA, 20 µg/mL), followed by nuclease inactivation at 75 °C for 10 min, consistent with established phage purification workflows [36,37].

Encapsidated phage DNA was released by treatment with proteinase K (100 µg/mL) in Tissue Lysis Buffer (ATL) at 56 °C for 20 min, followed by the addition of Lysis Buffer (AL) and 100% ethanol. DNA was purified using DNeasy Blood & Tissue spin columns (Qiagen, Hilden, Germany) according to the manufacturer’s protocol, including sequential washes with buffers AW1 and AW2 and elution in 50–100 µL Buffer AE. Because Oxford Nanopore sequencing requires relatively high DNA input, purified circular phage genomes were amplified by rolling-circle amplification (RCA) prior to library preparation. Among four independent cultures being induced, three (IPh_S1–IPh_S3) yielded phage DNA of sufficient quantity and quality for RCA and library preparation and are reported here; the fourth was excluded at the DNA yield stage and was not sequenced. All subsequent analyses are therefore based on three biological replicates. Libraries were sequenced on the Oxford Nanopore platform, and the resulting reads were quality-filtered, trimmed, and *de novo* assembled using the Oxford Nanopore Technologies (Oxford, UK). Consensus sequences were polished with the Medaka algorithm. Assembled reads from each biological replicate were aligned to the *D. vulgaris* reference chromosome (NC_002937.3) with PHASTEST to identify regions of enriched read coverage corresponding to excised prophage loci.

### 2.4. Comparative Genomic Analysis

Genomic coordinates of the seven PHASTEST-predicted prophage regions were overlaid with the sequencing alignment coordinates obtained from each induced phage replicate on a common chromosomal coordinate system (Unipro EGENE v53.1). The extent of overlap between predicted prophage boundaries and experimentally recovered sequencing reads was calculated at both the chromosome and locus levels. Based on the degree of concordance, each predicted prophage region was classified as experimentally validated (coincident with an excision hotspot), partially supported, or not detected.

### 2.5. Design, Assembly, and Sequence Validation of Synthetic Prophage Genomes

The 37.4 kb Region 1 prophage locus (chromosomal coordinates 233,143–270,532; 37,389 bp) was selected as the template for synthetic reconstruction. The region was partitioned into four overlapping long-range PCR fragments (Fragments A, B, C, and D) designed with ~50 bp overlaps between adjacent fragments to enable scarless isothermal DNA assembly; the overlap between Fragment A and the terminal fragment was designed to leave the product linear. The three invariant fragments A–C span 233,143–261,623 and constitute the conserved backbone of all constructs, while the terminal fragment D begins at 261,624 and defines the extent of the lysogeny control region retained. Three alternative terminal fragments were designed: D1 (7.6 kb, terminating at 269,223), D2 (4.1 kb, terminating at 265,723) and D3 (261 bp, terminating at 261,884; terminal overlap only). These yielded genomes of 36.1 kb (Construct C1), 32.6 kb (Construct C2) and 28.7 kb (Construct C3), corresponding to deletions of 1309, 4809 and 8648 bp from the 3′ end of the prophage.

Fragments were amplified from *D. vulgaris* genomic DNA by long-range PCR. Because the elevated GC content of the target locus (62.91%) was not efficiently amplified using Phusion high-fidelity DNA polymerase (NEB, Ipswich, MA, USA), amplification was instead performed using Q5 high-fidelity DNA polymerase (NEB, Ipswich, MA, USA) with primer-specific gradient annealing temperatures optimized for each fragment. PCR products were purified by agarose gel electrophoresis followed by gel extraction. The complete sequences of each fragment (A–C, and the D1/D2 variants) were verified by Oxford Nanopore sequencing, whereas the short D3 fragment was confirmed by Sanger sequencing. Sequencing reads were quality-filtered, trimmed, de novo assembled into a consensus sequence, polished using the Medaka algorithm, and aligned to the predicted Region 1 reference sequence to confirm sequence identity and integrity.

Fragment concentrations were determined and molar concentrations calculated based on fragment length and DNA mass. All fragments were normalized to 5 nM. For assembly, equimolar amounts of each fragment (5 fmol, 1 µL of 5 nM stock per fragment) were combined with NEBuilder HiFi DNA Assembly master mix (NEB, Ipswich, MA, USA), containing a 5′→3′ exonuclease, DNA polymerase, and DNA ligase, in a final reaction volume of 20 µL. Assembly reactions were incubated at 50 °C for 60 min followed by storage on ice. Assembled products were resolved on 0.4% agarose gels in 1× TAE buffer at 3 V/cm for 5 h. Bands corresponding to full-length assembled genomes were excised and purified using the QIAEX II Gel Extraction Kit (Qiagen, Hilden, Germany).

Purified synthetic genome constructs were sequenced by Oxford Nanopore long-read sequencing to a depth sufficient to span the full length of each ~29–36 kb construct. Reads were custom-aligned to the native Region 1 reference sequence (37,389 bp) using standard long-read alignment algorithms, and alignment coverage (*.bam files) was visualized in Unipro UGENE (Unipro LLC, Novosibirsk, Russia) to confirm construct length, assembly junction fidelity, and the absence of unintended rearrangements or sequence loss.

### 2.6. Electroporation, UV Induction, and Phenotypic Assays

Log-phase *D. vulgaris* cultures (100 mL, grown to OD_600_ ≈ 0.3) were rendered electrocompetent by three sequential washes in ice-chilled washing buffer (30 mM Tris-HCl, pH 8.0) [38], with the suspension volume reduced successively to 50 mL, 5 mL, and 1 mL. Aliquots of 50 µL competent cells were mixed with purified synthetic phage genome DNA (Constructs C1, C2, or C3) on ice for 10 min, transferred to a 1 mm-gap electroporation cuvette, and electroporated at a 1700 V instrument setting (measured actual output ≈ 1400 V, 1.4 ms) using an Eppendorf Eporator 2510 (Eppendorf, Hamburg, Germany).

Following electroporation, cells were recovered anaerobically for 3 h in N_2_/CO_2_-purged CSBK medium and subsequently exposed to UV irradiation for 1 min using a handheld 254 nm UV lamp positioned 20 cm above the culture surface to activate the bacterial SOS response and induce excision/expression of the electroporated prophage genome. Cultures were incubated anaerobically overnight (≥20 h; see qPCR results below), after which phage-containing supernatants were separated from cells by centrifugation (15,000 rpm, 10 min) followed by filtration through 0.22 µm syringe filter. Phage particles were concentrated by polyethylene glycol (PEG)/NaCl precipitation (10% PEG-8000/1 M NaCl), overnight at 4 °C.

Biological activity of the recovered phage was evaluated by spot assay. Constructs C1, C2 and C3; without electroporation; and a mock electroporation carried out without added DNA; and each was applied both to untreated and to UV-irradiated cultures. Briefly, 2 µL of concentrated phage preparation from each construct was spotted onto freshly prepared anaerobic *D. vulgaris* bacterial lawns, with ampicillin as a positive control for growth inhibition, phage buffer (PB) and untreated lawns as a negative control. Plates were incubated anaerobically for 72 h and examined visually and by bright-field microscopy to assess plaque morphology, including clear zones, turbid zones, and background lawn morphology (10 µm scale).

### 2.7. RNA Extraction, Reverse Transcription, and Quantitative PCR

Total RNA was extracted from UV-induced and untreated *D. vulgaris* cultures, which contained no synthetic prophage genome, at 0, 1, 2, 3, 4, and 21 h following UV exposure using the Monarch Total RNA Miniprep Kit (NEB, Ipswich, MA, USA), followed by on-column DNase treatment to remove residual genomic DNA. Sampling times were chosen to address two questions. The 0–4 h series was intended to capture the immediate SOS response and any early prophage transcription; the 21 h timepoint corresponds to the overnight incubation used for phage recovery in the induction experiments (Section 2.3) and was included to establish whether prophage transcripts were elevated at the point of harvest. Intermediate times between 4 and 21 h were not sampled because anaerobic sampling requires glovebox access and that interval fell outside the period during which the cultures could be handled without compromising anaerobic metabolism. Purified RNA was reverse-transcribed into cDNA using the RevertAid First Strand cDNA Synthesis Kit (Thermo Fisher Scientific, Waltham, MA, USA) using random hexamers, as appropriate for non-polyadenylated bacterial transcripts.

Four candidate reference genes (*recA*, *rpoB*, *atpD*, and *glnA*) were evaluated for expression stability across all treatment conditions using the BestKeeper, NormFinder, geNorm, and the comparative ΔCt algorithms implemented via the RefFinder web tool. *rpoB* was identified as the most stably expressed reference gene and used for normalization in all subsequent analyses (Figure S1).

Quantitative PCR was performed on cDNA using gene-specific primer pairs targeting five representative prophage structural/lytic genes (head capsid, tail, holin, portal, and tail tube) and the *rpoB* reference gene. Reactions were carried out using SYBR Green chemistry on a Bio-Rad CFX Opus real-time PCR system (Bio Rad, Hercules, CA, USA). No-reverse-transcriptase controls were included for each sample to verify the absence of genomic DNA contamination. Relative expression abundance was calculated using the comparative 2^−ΔΔCT^ method, where ΔCT = CT(target gene) − CT(*rpoB*), and ΔΔCT = ΔCT(treated) − ΔCT(untreated, 0 h calibrator), and fold-change reported as 2^−ΔΔCT^. All qPCR reactions were performed in biological replicates, and error bars in the reported data represent the standard deviation across replicates.

## 3. Results

### 3.1. Bioinformatic Identification of Prophage Regions Within the Desulfovibrio vulgaris Genome

To establish a genomic framework for synthetic phage engineering, we first performed a bioinformatic survey of prophage elements within the *Desulfovibrio vulgaris* Hildenborough genome. Prophages represent bacteriophage genomes integrated into bacterial chromosomes that can retain functional modules required for excision, replication, and virion assembly following induction, while also contributing to bacterial evolution, horizontal gene transfer, and host adaptation [18,19,20]. Consequently, accurate identification of these endogenous viral elements is an essential prerequisite for selecting candidate regions for synthetic reconstruction.

Prophage regions were predicted using PHASTEST, an automated platform that integrates sequence homology searches, viral protein annotation, genomic architecture, and prophage-specific structural signatures to identify and classify bacteriophage-derived genomic regions [21,22]. PHASTEST represents the latest generation of prophage prediction algorithms and provides improved accuracy and computational speed over earlier PHAST and PHASTER implementations [21,22]. The complete chromosome sequence was submitted in FASTA format to the PHASTEST server, where BLAST-based similarity searches were performed against a curated bacteriophage genome database. Predicted regions were assigned to one of three confidence categories according to the PHASTEST scoring algorithm [22]. Intact prophages contain a complete complement of genes required for a functional viral life cycle, including structural, DNA replication, packaging, and host lysis modules. Questionable prophages retain numerous phage-associated genes but lack one or more essential functional components, whereas incomplete prophages represent highly degraded remnants that have undergone extensive gene loss during long-term evolution [18,39]. To visualize prophage organization across the chromosome, genomic annotations were rendered as both chromosome-wide circular maps and high-resolution linear gene maps.

**Figure 1:**
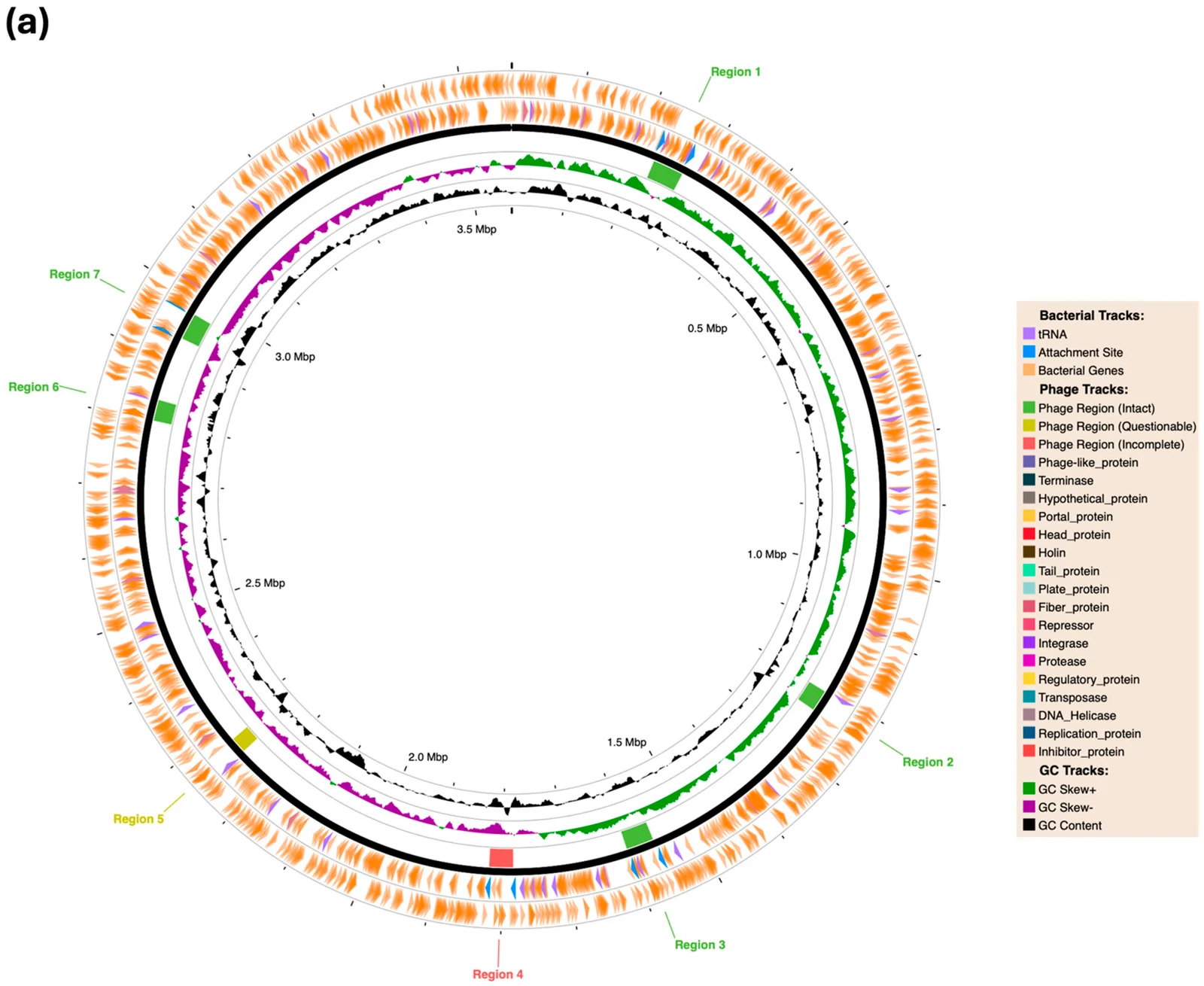

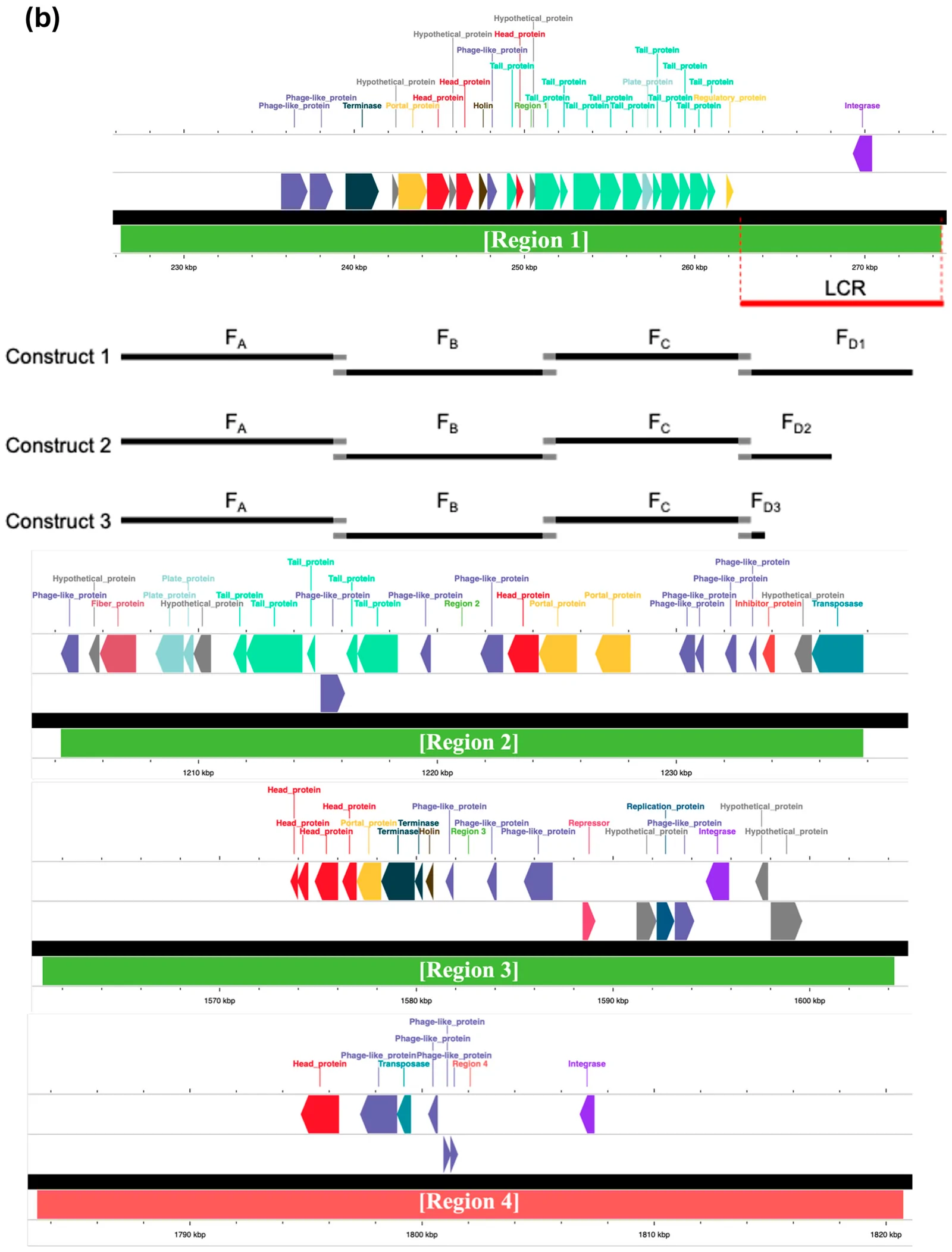

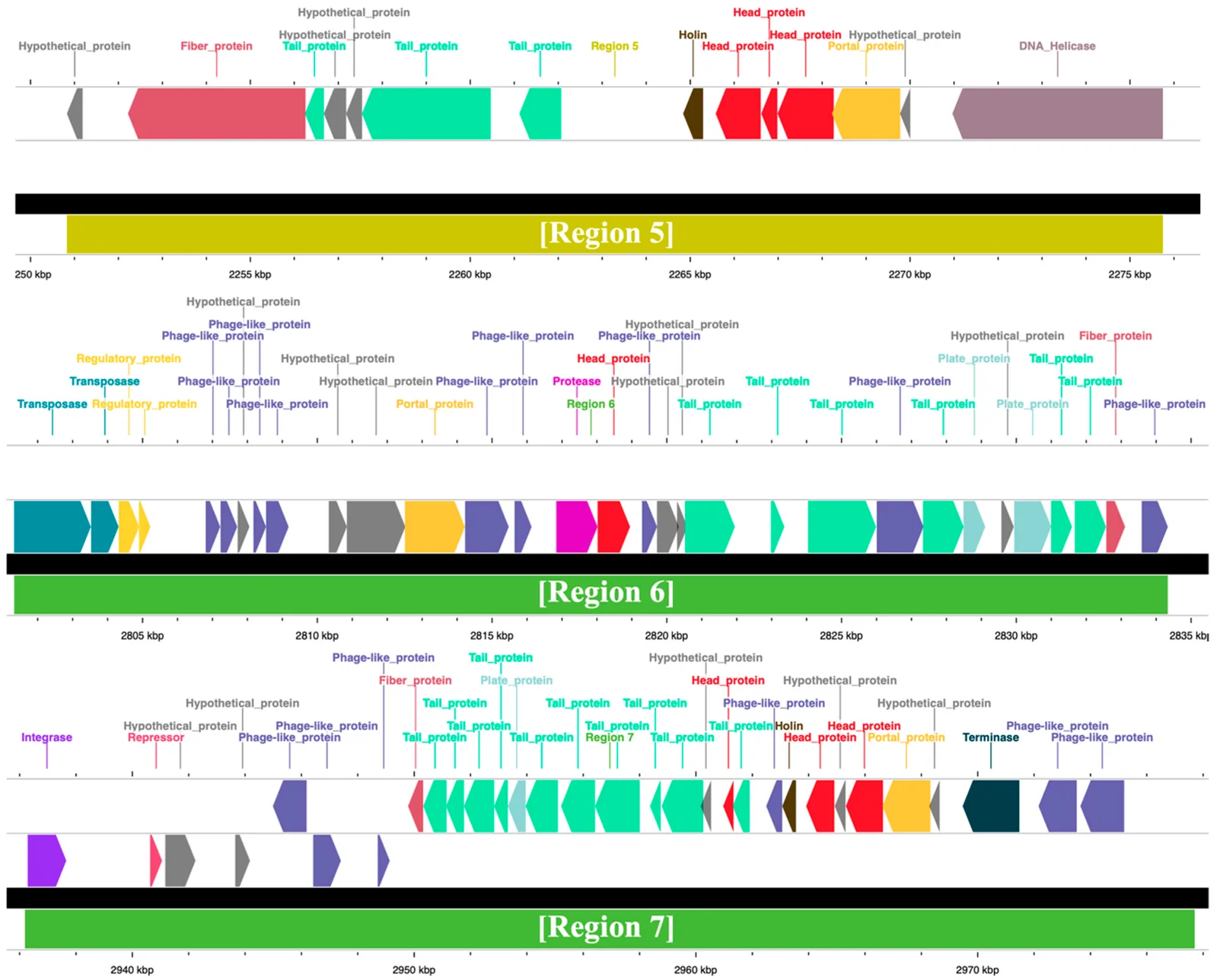
Bioinformatic prediction of prophage regions across the *Desulfovibrio vulgaris* chromosome using PHASTEST. (**a**) Circular genome map showing bacterial and phage-associated coding sequences, tRNA genes, attachment sites, and the seven predicted prophage regions (colored by completeness classification), overlaid with local GC content and GC skew tracks (see legend). (**b**) High-resolution linear maps of the seven predicted prophage regions (Region 1–7), annotated with structural and lytic gene modules (integrase, repressor, terminase, portal, head, tail, and related proteins) and colored by PHASTEST completeness classification (intact, questionable, or incomplete; corresponding to Table 1), with Region 1 showing the boundaries of PCR fragments A–D and the deletion breakpoints of constructs C1–C3 against the annotated linear gene map.

PHASTEST analysis identified seven prophage-like regions distributed throughout the 3,570,858 bp chromosome of *D. vulgaris* (Figure 1). Among the 3184 predicted coding sequences within the genome, 159 genes were annotated as phage-derived, while the remaining 3025 genes comprised the core bacterial chromosome. Five of the seven regions were classified as intact, one as questionable, and one as incomplete (Table 1), indicating that *D. vulgaris* harbors an unusually rich repertoire of endogenous prophages with varying degrees of structural preservation. The abundance of integrated prophages is consistent with previous observations that lysogeny is widespread among environmental bacteria and contributes substantially to microbial genome diversification [18,25].

The chromosome-wide distribution of these prophages is illustrated in Figure 1a. The outer concentric rings depict the organization of bacterial coding sequences, whereas the inner annotation tracks identify phage-derived genes grouped according to their predicted functional categories, including integrases, terminases, portal proteins, capsid proteins, tail proteins, holins, lysins, and regulatory proteins. Candidate prophage regions are highlighted according to their PHASTEST classification (intact, questionable, or incomplete), providing a genome-scale overview of putative viral integration sites.

To further examine genomic features associated with prophage insertion, GC content and GC skew were analyzed across the chromosome using a 1 kb sliding window. The GC content track (black histogram) together with the GC skew profile (green and purple histogram) revealed localized transitions in nucleotide composition coinciding with several prophage boundaries (Figure 1a). Such compositional shifts are commonly associated with horizontally acquired DNA and are consistent with the evolutionary integration of bacteriophage genomes into bacterial chromosomes [18,39].

Detailed gene organization for each predicted prophage is presented in the linear annotation maps (Figure 1b). These maps demonstrate that the intact regions exhibit the canonical modular organization characteristic of temperate bacteriophages, including integration, DNA replication, DNA packaging, head assembly, tail morphogenesis, host lysis, and transcriptional regulation modules [18]. In contrast, the questionable and incomplete regions display fragmented gene organization and missing structural modules, suggesting progressive genomic decay during long-term residence within the bacterial chromosome.

The structural characteristics of each predicted prophage are summarized in Table 1. Regions 1, 2, 3, 6, and 7 were classified as intact prophages, with PHASTEST completeness scores ranging from 140 to 150, well above the intact-prophage threshold of >90. These regions ranged from 33.0 to 43.6 kb in length and encoded 40–51 predicted proteins, consistent with relatively complete temperate phage genomes. Region 5 received a score of 90, placing it at the boundary of the questionable category. This region spans 24.9 kb and encodes 25 predicted proteins, suggesting partial loss of functional modules. Region 4 was classified as an incomplete prophage with a score of 60. Although its genomic length (37.3 kb) is comparable to that of several intact prophages, it encodes only 15 predicted proteins, indicating substantial gene loss and structural degradation during evolution.

Despite differences in structural completeness, all seven prophage regions exhibited GC contents ranging from 58.4% to 65.6%, closely matching the average GC content of the *D. vulgaris* chromosome (Table 1). This limited variation suggests that these prophages have undergone extensive nucleotide amelioration during prolonged association with their bacterial host, consistent with their long-term evolutionary integration [18]. The high prevalence of structurally intact prophages, together with the preservation of complete structural and lytic gene modules, identified Regions 1, 2, 3, 6, and 7 as the most promising candidates for subsequent induction experiments and synthetic phage engineering.

### 3.2. Chromosomal Mapping of UV-Induced Desulfovibrio vulgaris Temperate Phages

To experimentally validate the prophage regions predicted by PHASTEST, endogenous temperate phages were induced from *D. vulgaris* cultures by UV irradiation. UV-induced DNA damage activates the bacterial SOS response, a well-established trigger of prophage excision and entry into the lytic cycle in many temperate bacteriophages [19,20,40]. Four independent 0.2 L cultures were induced under identical conditions to promote prophage excision and phage production. Following induction, phage particles were purified from culture supernatants using nuclease treatment to eliminate contaminating host DNA and RNA prior to viral genome isolation, following established phage purification strategies [36,37]. Purified phage DNA was subsequently amplified by rolling-circle amplification (RCA) and subjected to whole-genome sequencing.

**Figure 2:**
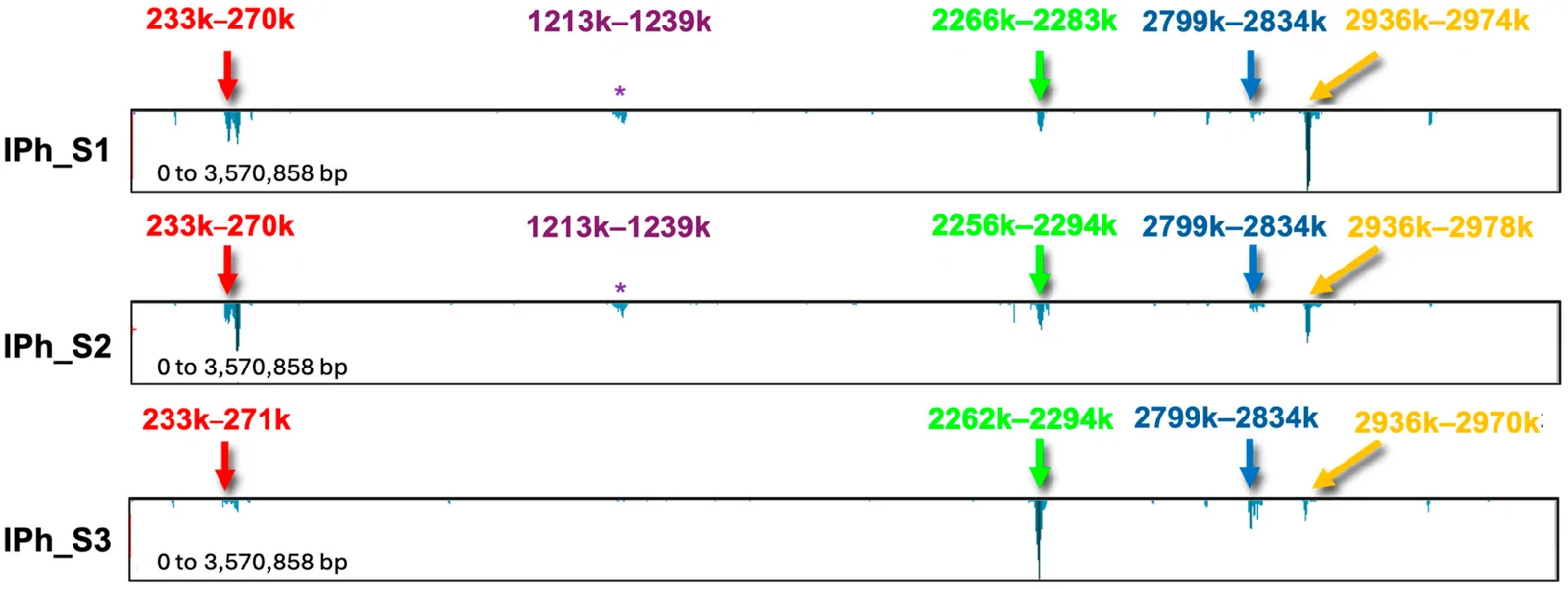
Whole-genome mapping of recurrent prophage excision hotspots following UV induction of *Desulfovibrio vulgaris*. Sequencing reads obtained from purified phage DNA (replicates IPh_S1–IPh_S3) were aligned to the *D. vulgaris* Hildenborough reference chromosome (NC_002937.3). Four recurrent excision hotspots were consistently detected across independent biological replicates, indicated by red, green, blue and yellow arrows, and corresponded to Regions 1, 5, 6 and 7, respectively. A lower-frequency excision event observed predominantly in samples IPh_S1 and IPh_S2 (purple asterisk) corresponds to Region 2. The high reproducibility of the major hotspots provides experimental validation of the PHASTEST-predicted intact prophage regions shown in Figure 1.

Sequencing reads from each biological replicate (IPh_S1–IPh_S3) were aligned against the *D. vulgaris* Hildenborough reference chromosome (NCBI accession NC_002937.3) to identify genomic regions corresponding to excised prophage genomes. Whole-genome sequencing is an effective approach for reconstructing viral genomes and mapping structural genomic elements with high accuracy [41,42,43]. Alignment profiles revealed multiple discrete regions of highly enriched read coverage, representing recurrent prophage excision hotspots (Figure 2). Four excision hotspots were recovered in all three biological replicates, corresponding to Regions 1, 5, 6 and 7. A fifth locus, Region 2, was recovered in IPh_S1 and IPh_S2 but not in IPh_S3, and Regions 3 and 4 were not recovered from any replicate. Sequencing depth for each replicate is given in Table S1. The concentration of reads at a small number of discrete loci, rather than across the chromosome, indicates that UV irradiation activates specific endogenous prophages rather than producing random chromosomal fragmentation.

Importantly, these experimentally identified excision hotspots corresponded to the intact prophage regions independently predicted by PHASTEST (Section 3.1), even though defined 2256–2294 kb region is recognized as questionable in completeness. This observation provides orthogonal validation of the computational prophage annotation and prediction versus inducibility discordance. The integration of bioinformatic prophage prediction with experimental validation has proven effective for distinguishing biologically active prophages from cryptic prophage remnants [18,25]. The concordance between bioinformatic prediction and whole-genome sequencing confirms that the majority of PHASTEST-classified intact regions are biologically inducible and capable of excising from the bacterial chromosome under DNA damage conditions.

Because the three cultures were derived from the same clonal stock, this variability cannot reflect genetic differences between them. Prophage induction is instead a stochastic, single-cell decision: under uniform stress only a fraction of the population commits to the lytic cycle, and that fraction differs between prophages according to the sensitivity of their regulatory circuits [19,20,44]. It is further modulated by the dose individual cells receive, since 254 nm light is strongly attenuated at the optical density used and stirring exposes cells intermittently rather than uniformly. Loci induced in the smallest fraction of cells are therefore the least reproducibly recovered, and the inconsistent detection of Regions 2 is best interpreted as a low and variable induction frequency rather than absence from the genome; sequencing statistics for each replicate are given in Table S1.

Collectively, these genome-wide sequencing data demonstrate that UV induction reproducibly triggers excision of multiple endogenous prophages from the *D. vulgaris* chromosome. These findings are consistent with earlier reports demonstrating inducible temperate phages in *Desulfovibrio* species [15,16,45]. The strong agreement between computational prophage prediction and experimentally mapped excision boundaries established a robust framework for selecting intact, inducible prophage regions for downstream synthetic genome reconstruction and engineering.

### 3.3. Comparison of Predicted Prophages and Empirically Induced Viral Genomes

**Figure 3:**
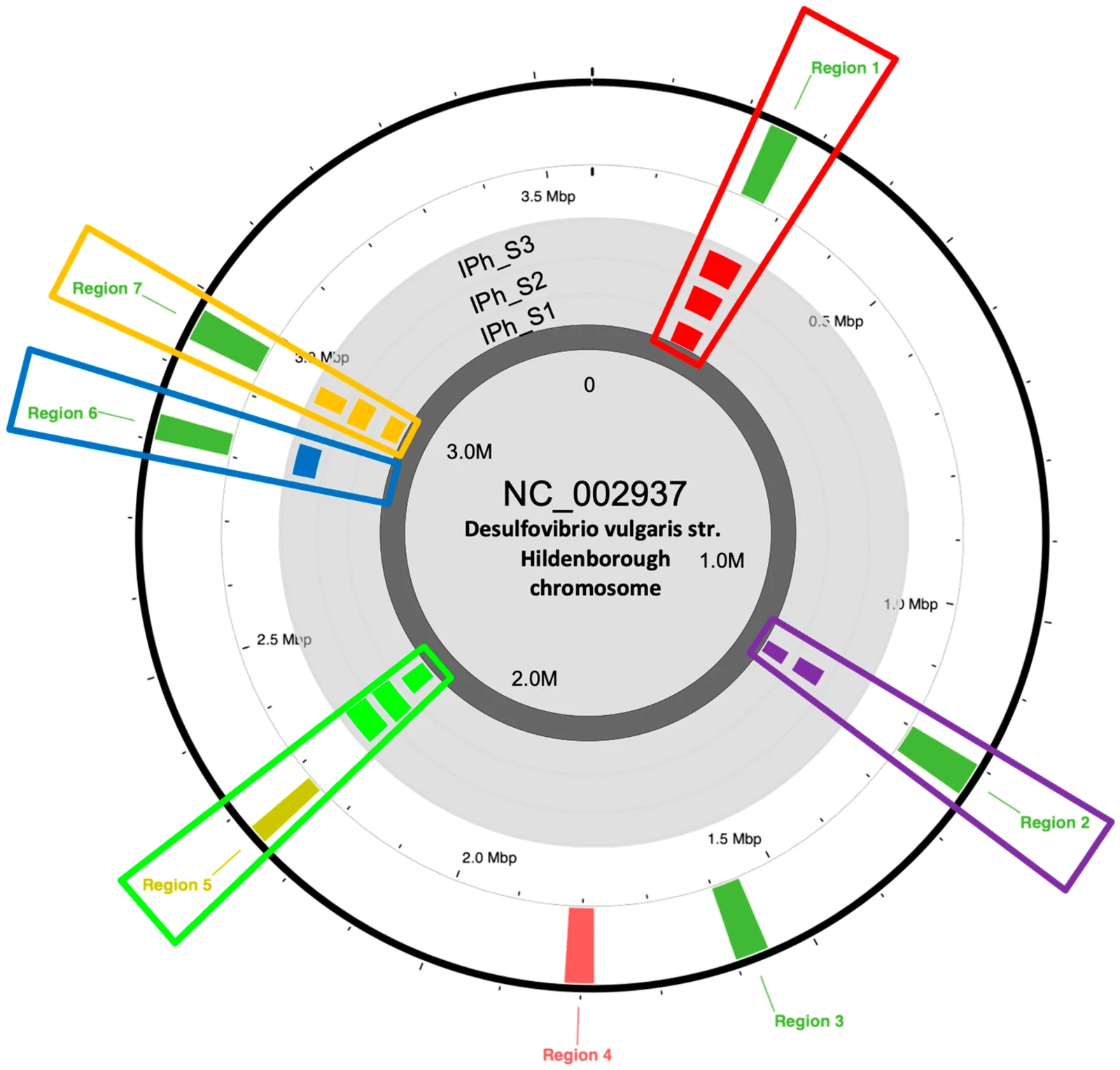
Comparative alignment of bioinformatically predicted and empirically induced *Desulfovibrio vulgaris* prophage genomes. Circular chromosome map showing the spatial relationship between the seven PHASTEST-predicted prophage regions (outer track) and sequencing alignments from three independently induced phage preparations (IPh_S1–IPh_S3; inner concentric tracks).

To evaluate the accuracy of the bioinformatic prophage predictions, the coordinates of the seven PHASTEST-identified prophage regions (Section 3.1) were directly compared with the genomic locations of viral sequences recovered from UV-induced phage particles (Section 3.2). Predicted prophage boundaries and experimentally derived sequencing alignments were integrated onto a common chromosomal framework to enable both chromosome-scale and locus-specific comparisons (Figure 3).

The resulting comparative analysis revealed strong agreement between computational prediction and experimental induction. Of the five regions classified as intact prophages by PHASTEST, three (Regions 1, 6 and 7) were recovered from induced phage particles in all three biological replicates (IPh_S1–IPh_S3) and one (Region 2) in two of the three, indicating that these loci are actively inducible and capable of generating packaged viral genomes following UV exposure. Region 3 was not recovered from any replicate. Region 5, classified by PHASTEST as questionable, was recovered in all three replicates and was among the most consistently represented loci in the dataset. Prophage inducibility therefore correlated only partially with computational completeness, and the discordance was observed in both directions: an intact region that was not induced, and a questionable region that was. However, the high concordance between computational prediction and experimental validation demonstrates the effectiveness of modern prophage discovery pipelines for identifying biologically functional prophages [21,22].

A notable observation was the strong experimental support for Region 5, which had been classified by PHASTEST as a questionable prophage. Despite receiving a lower confidence score than the intact regions, Region 5 consistently generated sequencing signals in induced phage preparations and aligned with one of the major excision hotspots identified in Figure 2. This finding demonstrates that prophage functionality cannot always be inferred solely from computational completeness scores and suggests that Region 5 retains sufficient genetic components to support excision and viral genome packaging. Similar discrepancies between computational annotation and biological activity have been reported in other bacterial systems, emphasizing the importance of experimental validation for distinguishing active prophages from inactive genomic remnants [18,25].

In contrast, Region 2, which was classified as an intact prophage by PHASTEST, was recovered in only two of the three replicates, being absent from IPh_S3. Alignment signals associated with this region were detected at substantially lower and more variable frequency than those observed for Regions 1, 5, 6 and 7, suggesting either reduced inducibility under UV-mediated DNA damage conditions or lower phage production efficiency. These results indicate that structural completeness alone does not necessarily predict the magnitude of prophage activation, as prophage induction is influenced by host regulatory circuits and environmental conditions in addition to genome integrity [19,20].

Similarly, Region 3, despite being categorized as an intact prophage, showed little or no detectable overlap with the induced viral sequencing reads. The absence of a corresponding excision hotspot suggests that this region remained transcriptionally inactive or did not undergo excision under the induction conditions used in this study. While the genomic architecture of Region 3 appears largely intact, its activation may require alternative environmental stimuli or regulatory conditions not examined here, as different temperate phages respond to distinct stress signals and induction pathways [19].

Consistent with its classification as an incomplete prophage, Region 4 showed no detectable correspondence with the induced viral genome datasets. The absence of sequencing enrichment at this locus supports the conclusion that Region 4 represents a highly degraded prophage remnant lacking the genetic capacity necessary for productive excision and viral propagation, a characteristic commonly observed for cryptic prophages that have accumulated mutations during long-term residence within bacterial chromosomes [18,39].

Figure 3 summarizes the chromosome-wide relationship between predicted prophage regions and experimentally recovered viral genomes. The concentric inner tracks represent sequencing alignments from independent induced phage preparations, while the outer chromosome track indicates the locations of the seven PHASTEST-predicted prophage regions. Regions exhibiting reproducible overlap between prediction and experimental sequencing are readily apparent, demonstrating substantial concordance between the computational and empirical datasets. Collectively, these results validate most of the bioinformatically predicted prophages and identify Regions 1, 5, 6 and 7 as the most robustly inducible endogenous viral elements within the *D. vulgaris* genome. A quantitative summary of prophage coordinates, classification scores, overlap statistics, and experimental validation status is provided in Table 2. These analyses established a rational basis for selecting candidate prophage loci for subsequent synthetic genome reconstruction and engineering studies.

### 3.4. Construction of Synthetic Desulfovibrio vulgaris Prophage Genomes

Following identification of inducible prophage loci, Region 1 was selected as the template for synthetic phage engineering because it represented one of the most consistently induced and structurally complete prophages identified by both bioinformatic prediction and experimental validation (Figure 1, Figure 2 and Figure 3). To investigate the genetic requirements for phage production, a series of progressively truncated synthetic genomes was designed. The deletions target the prophage lysogeny control region, the interval lying between the last structural gene and the attachment site attR; PHASTEST places attL at 226,281–226,292 and attR at 274,490–274,501, with the integrase immediately inside attR, which is the canonical arrangement in temperate phage genomes. PHASTEST assigns a function to two of the seventeen open reading frames in this interval (Table 3), an integrase (PP_00230) and a CopG-family transcriptional regulator (PP_00214), while the remaining fifteen are annotated as hypothetical. Because the functions of most genes in the region are therefore unknown, the constructs were designed as a length series rather than as single-gene deletions, each retaining progressively less of the region, so that any dependence of phage behavior on the extent of the retained interval could be assessed. The structural, DNA packaging, replication and lysis modules lie upstream of this interval and are retained intact in every construct. Rational engineering of bacteriophage genomes through targeted deletion of nonessential or lysogeny-associated genes has emerged as an effective strategy for investigating phage biology and generating synthetic phages with altered biological properties [11,26,28].

**Figure 4:**
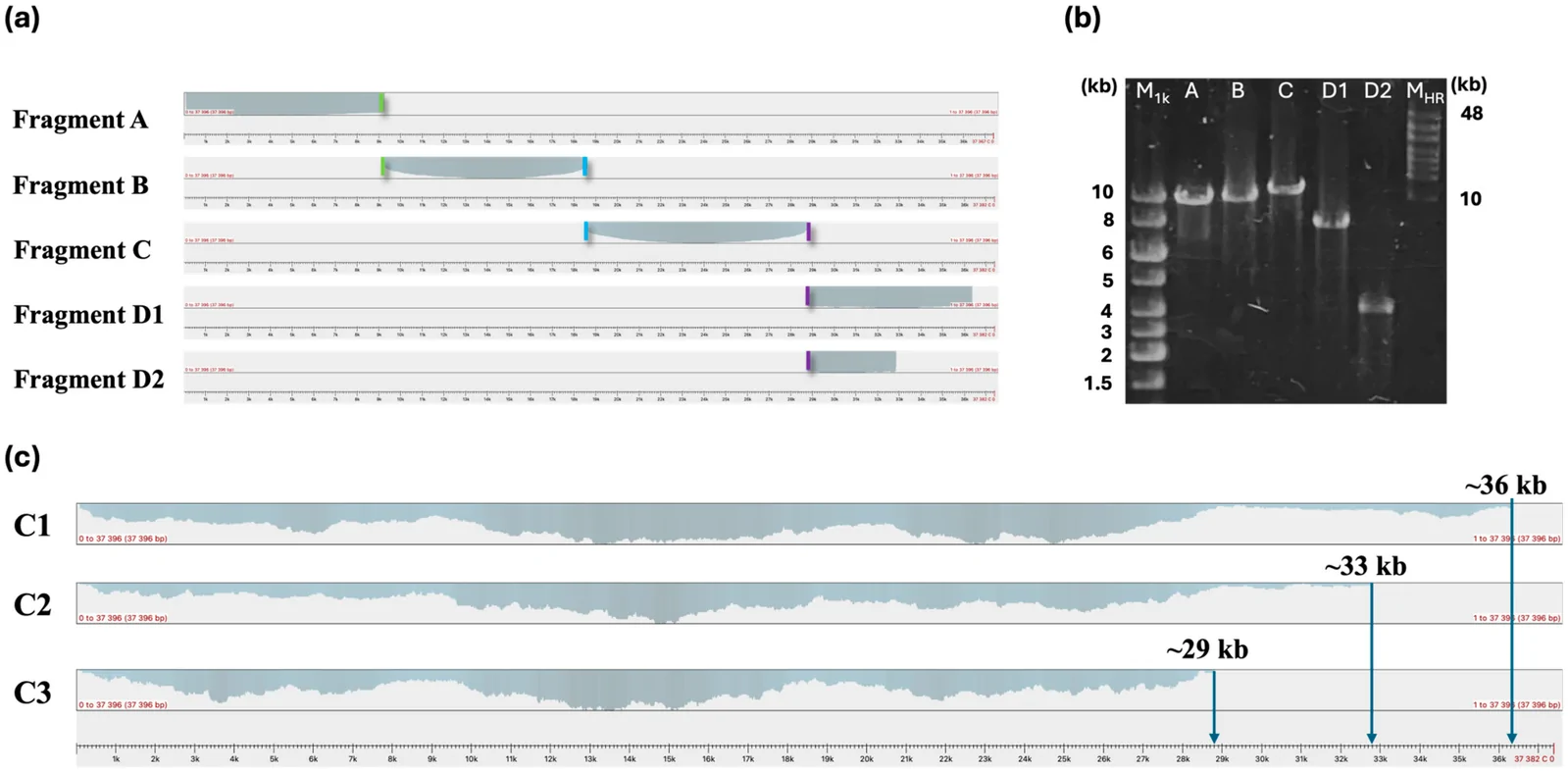
Construction and sequence validation of synthetic *Desulfovibrio vulgaris* Region 1 prophage genomes. (**a**) Design of four overlapping long-range PCR fragments (A–D) used for scarless assembly of synthetic prophage genomes. Three invariant fragments (A–C) were combined with alternative terminal Fragment D variants (D1–D3) to generate progressively truncated constructs; Oxford Nanopore sequencing confirmed fragment identity and overlap boundaries. (**b**) Agarose gel electrophoresis of purified PCR amplicons used for genome assembly (lanes A–D2; M, DNA size markers). (**c**) Nanopore sequencing coverage of assembled synthetic genomes aligned to the native Region 1 reference sequence. Constructs C1, C2, and C3 produced continuous alignments of approximately 36, 33, and 29 kb, respectively, with alignment endpoints coinciding with the engineered deletion boundaries, confirming accurate assembly.

The 37.4 kb Region 1 prophage genome was partitioned into four overlapping long-range PCR fragments to facilitate scarless isothermal DNA assembly (Figure 4a). Three invariant fragments (A, B, and C), each approximately 10 kb in length, constituted the conserved backbone of the synthetic genomes, whereas the terminal fragment (Fragment D) was generated in multiple versions containing progressively larger deletions. Fragment D1 retained a 7.6 kb terminal segment, Fragment D2 was shortened to 4.1 kb, and Fragment D3 consisted of a minimal 261 bp terminal overlap, thereby generating three synthetic prophage variants of decreasing genome size. The consequences at the gene level are summarized in Table 3. Construct C1 removes the integrase in its entirety, together with the 3′ 63 bp of the adjacent PP_00229, and retains the remainder of the lysogeny control region, including the CopG-family regulator. Construct C2 additionally removes PP_00222 to PP_00229 and the 3′ 46 bp of PP_00221, but retains PP_00214 to PP_00220 and therefore the regulator. Construct C3 removes the region in full: PP_00215 to PP_00230 are deleted and only the first 10 bp of PP_00214 remain, which cannot encode a functional product. The integrase is thus absent from all three constructs, whereas the single annotated regulatory gene distinguishes C1 and C2, which retain it, from C3, which does not. This modular assembly strategy follows established synthetic biology approaches for constructing large bacteriophage genomes from overlapping DNA fragments [26,29,30].

Prior to assembly, each PCR amplicon was verified by Oxford Nanopore sequencing, while the shortest D3 fragment was confirmed by Sanger sequencing. Alignment of the sequencing reads against the designed reference sequences demonstrated complete agreement with the intended fragment architectures, including accurate terminal overlap regions required for seamless assembly (Figure 4a). Long-read nanopore sequencing provides an effective platform for validating large synthetic DNA constructs because individual reads frequently span entire assembly junctions, allowing direct verification of genome integrity [41,42,43]. Gel electrophoresis further confirmed the successful amplification and purification of each precursor fragment before genome reconstruction (Figure 4b).

Equimolar DNA fragments were subsequently assembled using a one-step isothermal assembly reaction to generate three synthetic prophage genomes. Isothermal DNA assembly has become a widely adopted method for scarless construction of large DNA molecules because it enables seamless joining of multiple overlapping fragments in a single reaction [46]. Assembly products were resolved by low-percentage agarose gel electrophoresis, and bands corresponding to full-length recombinant genomes were excised and purified for sequence verification.

To confirm the structural integrity of the assembled genomes, purified constructs were subjected to Oxford Nanopore sequencing and aligned against the native Region 1 prophage sequence. Continuous long-read coverage extended across the entire length of each construct, demonstrating successful assembly of the designed genomes (Figure 4c). The three recombinant genomes exhibited sizes of approximately 36 kb (Construct C1), 33 kb (Construct C2), and 29 kb (Construct C3), in excellent agreement with the predicted deletion designs.

Importantly, sequence alignment confirmed that each construct retained the expected genomic organization without evidence of rearrangements or unintended sequence loss. All fragment junctions were reconstructed precisely, producing seamless assemblies with complete sequence identity to the designed synthetic genomes. Progressive decreases in sequencing coverage termination points corresponded exactly to the engineered deletion boundaries, providing independent confirmation of the intended genome truncations (Figure 4c). Similar long-read sequencing strategies have become the standard for validating synthetic genomes and large recombinant DNA assemblies [41,42].

Collectively, these results demonstrate the successful reconstruction of three sequence-verified synthetic *D. vulgaris* prophage genomes representing progressively reduced versions of the native Region 1 chromosome. These validated synthetic genomes served as the starting materials for subsequent electrotransformation experiments designed to evaluate phage activation, genome replication, and particle production in *D. vulgaris*. The successful reconstruction of these large endogenous prophage genomes using a modular, scarless assembly strategy establishes a practical platform for future genome engineering and synthetic phage development in anaerobic bacteria [27,28,29].

### 3.5. Phenotypic Evaluation of Synthetic Prophage Genomes Following Introduction into Desulfovibrio vulgaris

**Figure 5:**
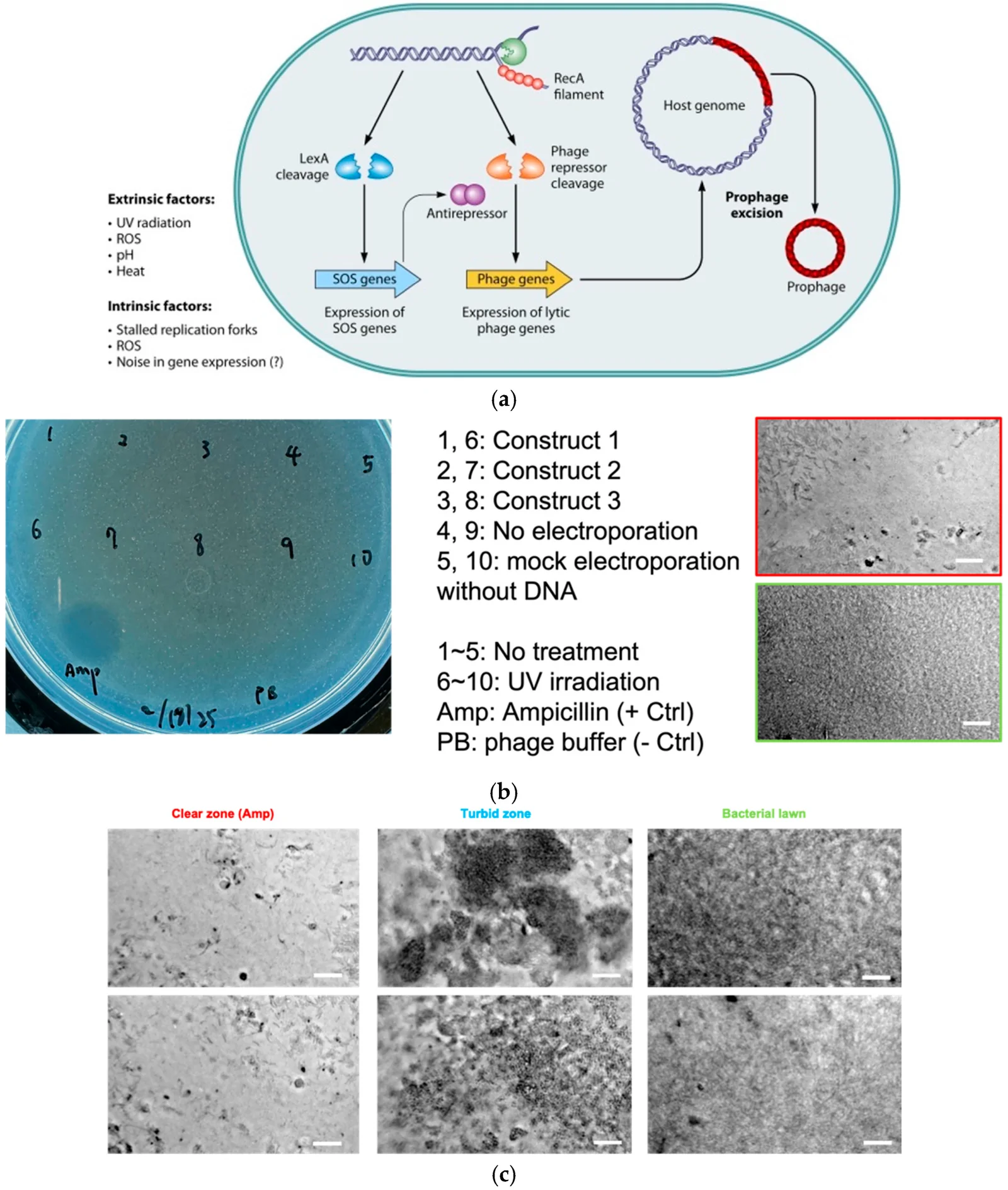
Phenotypic evaluation of engineered synthetic prophage genomes following introduction into *Desulfovibrio vulgaris*. (**a**) Experimental workflow: electroporation of synthetic prophage genomes, UV-mediated activation of the SOS response, prophage induction, and recovery of phage-containing culture supernatants for downstream assays. (**b**) Spot assay on anaerobic *D. vulgaris* lawns using concentrated phage preparations from Constructs C1–C3, with ampicillin as a positive control. The antibiotic produced a clear inhibition zone, whereas all three synthetic phage preparations generated localized turbid regions without classical plaque formation. (**c**) Bright-field micrographs comparing cell morphology within the ampicillin inhibition zone (**left**), the synthetic phage-induced turbid region (**center**), and the untreated bacterial lawn (**right**), showing increased turbidity and refractile extracellular material at phage application sites. Scale bars, 10 μm.

To determine whether the engineered prophage genomes retained biological activity following synthetic reconstruction, the three sequence-verified constructs (C1–C3) were introduced into competent *Desulfovibrio vulgaris* cells by electroporation. Electroporation has become one of the most widely used approaches for introducing large DNA molecules into bacterial hosts, including synthetic bacteriophage genomes for phage engineering and rebooting studies [27,29,47]. Following recovery, transformed cultures were exposed to UV irradiation to activate the bacterial SOS response and induce excision and expression of the engineered prophage genomes [19,40] (Figure 5a). Culture supernatants containing released phage particles of each synthetic construct were subsequently collected, concentrated, and evaluated for biological activity using a spot assay on freshly prepared anaerobic *D. vulgaris* lawns and incubated anaerobically for 72 h. Ampicillin was included as a positive control for growth inhibition, whereas untreated bacterial lawns served as negative controls (Figure 5b). As expected, the ampicillin control produced a well-defined zone of growth inhibition, confirming the suitability of the assay for detecting localized antibacterial activity.

In contrast, none of the three synthetic phage preparations produced the large, transparent plaques typically associated with productive lytic infection [10,18]. Instead, all three constructs consistently generated localized turbid zones at the application sites (Figure 5b). These regions were readily distinguishable from both the surrounding confluent bacterial lawn and the antibiotic-induced inhibition zone, indicating that the engineered phage genomes elicited a reproducible but noncanonical phenotypic response. The buffer-only control and the mock-electroporated in both untreated and UV-irradiated supernatant produced no zone distinguishable from the surrounding lawn, indicating that neither buffer containing the induced phages itself nor UV-induced release of endogenous prophage was sufficient, under these conditions, to produce the localized turbid phenotype.

Bright-field microscopy further revealed marked differences in the cellular architecture of the three assay regions (Figure 5c). The ampicillin-treated area contained only sparse residual cells, consistent with efficient growth inhibition, whereas untreated control regions displayed a dense and homogeneous bacterial lawn. In comparison, the synthetic phage application sites exhibited increased local turbidity together with the accumulation of highly refractile extracellular material, producing a heterogeneous microstructure distinct from either the positive or negative controls. This phenotype was consistently observed for all three independently engineered constructs.

The absence of conventional plaque formation suggests that the synthetic genomes did not produce efficient lytic propagation under the experimental conditions used in this study. Temperate phages frequently exhibit incomplete induction or pseudolysogenic behaviors that differ from the clear-plaque phenotypes produced by obligately lytic phages [18,20,44]. Nevertheless, the reproducible formation of localized turbid zones indicates that introduction and UV induction of the engineered prophage genomes altered the physiological response of the bacterial population. Whether these changes reflect limited phage replication, incomplete lytic development, altered host physiology, or another phage–host interaction cannot be determined from the present assay alone.

Collectively, these observations demonstrate that the engineered synthetic prophage genomes generate reproducible biological effects in *D. vulgaris* following electroporation and UV induction, although these effects differ from the classical plaque morphology expected for fully lytic bacteriophages. Similar observations have been reported during early stages of synthetic phage engineering, where successful genome reconstruction does not necessarily result in efficient phage propagation or plaque formation until additional host factors or optimized induction conditions are identified [27,28,29]. As an additional proof-of-concept, we attempted to engineer the Region 1 synthetic prophage by incorporating a GFP reporter to develop a phage-based biosensor for *D. vulgaris*. Despite successful genome assembly, no detectable reporter expression or functional phage-mediated signal was observed following transformation and induction. These results suggest that additional phage functions required for productive infection, reporter expression, or particle formation may reside outside Region 1 or require coordinated interactions with other inducible prophage regions. Although unsuccessful, these experiments identified important technical limitations and provided valuable guidance for future development of engineered phage-based biosensors.

### 3.6. Quantitative PCR Analysis of UV-Induced Prophage Gene Expression

To establish an appropriate induction window for subsequent synthetic phage experiments, we characterized the temporal transcriptional response of representative prophage genes following UV-mediated induction in wild-type *Desulfovibrio vulgaris*, which contained no synthetic prophage genome. UV irradiation is a well-established trigger of the bacterial SOS response and prophage induction through DNA damage-mediated activation of temperate phages [18,19,40]. Quantitative real-time PCR (qPCR) was performed to monitor the expression of five conserved prophage genes encoding the head capsid, tail, holin, portal, and tail tube proteins over a 21 h induction period. Transcript abundance was quantified at 0, 1, 2, 3, 4, and 21 h following UV exposure using the housekeeping gene *rpoB* as the internal reference. Relative expression levels were calculated using the 2^−ΔΔCT^ method with the untreated 0 h sample serving as the calibrator.

**Figure 6:**
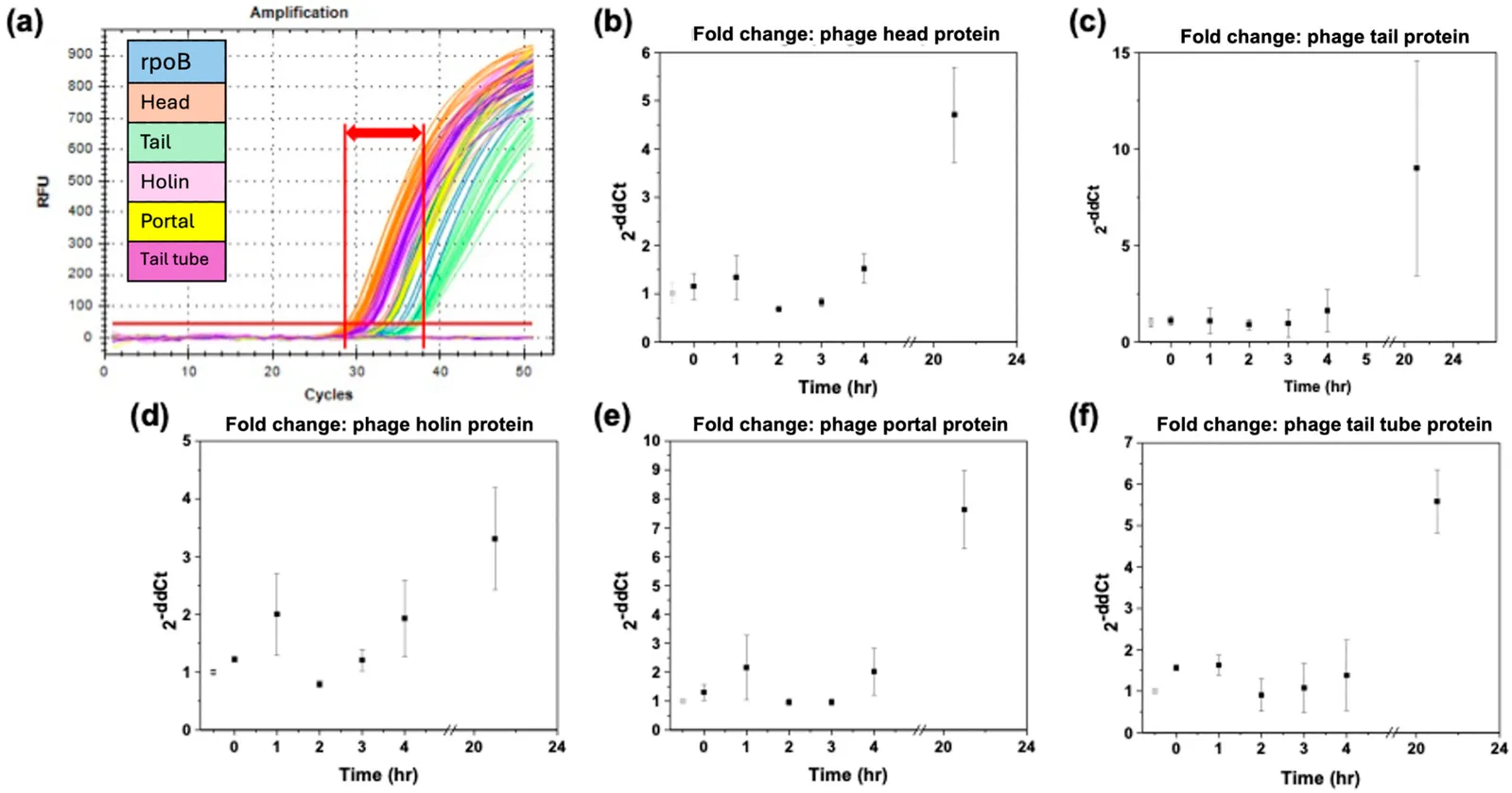
Quantitative PCR analysis of prophage gene expression following UV induction in wild-type *Desulfovibrio vulgaris*. (**a**) Representative amplification curves for the five prophage target genes and the *rpoB* reference gene. (**b**–**f**) Relative expression (2−ΔΔCT) of the head capsid, tail, holin, portal, and tail tube genes following UV induction, normalized to *rpoB* and expressed relative to the untreated 0 h control. Error bars represent the standard deviation across biological replicates. These cultures contained no synthetic prophage genome; the transcripts measured therefore derive from the endogenous chromosomal prophages.

Prior to transcript analysis, candidate reference genes were evaluated using four independent normalization algorithms, including BestKeeper, NormFinder, geNorm, and the comparative ΔCT method. Integrated stability ranking identified *rpoB* as the most stable reference gene across all experimental conditions (Figure S1), supporting its use for normalization throughout the study. Similar multi-algorithm approaches are widely recommended for selecting stable reference genes for quantitative gene expression analyses. Representative amplification curves demonstrated robust and reproducible amplification for all target genes, with cycle threshold (CT) values ranging from approximately 28 to 38 cycles (Figure 6a). The *rpoB* reference gene exhibited minimal variation across the complete time course, confirming stable expression following UV treatment.

Temporal expression analysis revealed a coordinated transcriptional response among the five prophage genes (Figure 6c–f). During the early induction period (0–4 h), transcript levels remained near baseline, exhibiting only modest increases of approximately 1–2-fold relative to untreated controls. In contrast, all targets showed pronounced upregulation after 21 h of induction, with transcript abundance increasing between approximately 3- and 10-fold depending on the gene examined. The largest increases were observed for the portal and tail genes, whereas the head, holin, and tail tube genes displayed more moderate but reproducible induction. Because the interval between 4 and 21 h was not sampled, these data do not resolve the time at which upregulation begins; they establish only that transcript levels were near baseline through 4 h and elevated by 21 h. Accordingly, 21 h is reported here as a timepoint at which late gene expression is reliably detectable, rather than as the onset of induction.

The coordinated late-stage activation of multiple structural and lytic genes is consistent with the well-characterized temporal transcriptional program of temperate bacteriophages, in which structural proteins, DNA-packaging components, and host lysis genes are expressed during the late phase of the lytic cycle following SOS-mediated prophage induction [18,20,44].

## 4. Discussion

Temperate bacteriophages represent an abundant yet largely untapped genetic resource in many environmentally and industrially important bacteria [18,20]. In SRBs such as *D. vulgaris*, endogenous prophages have received relatively little attention despite their potential influence on genome evolution, horizontal gene transfer, and host physiology [16,45]. In the present study, we established an integrated workflow that combines computational prophage prediction, experimental validation of inducible prophages, synthetic genome reconstruction, and functional characterization. This strategy enabled us to move beyond prophage annotation alone and experimentally evaluate the feasibility of engineering endogenous *D. vulgaris* phage genomes.

A major finding of this work is the strong agreement between computational prophage prediction and experimental induction. PHASTEST identified seven prophage-like regions distributed throughout the *D. vulgaris* chromosome, five of which were classified as intact based on their structural organization and gene content [21,22]. Whole-genome sequencing of UV-induced phage particles independently confirmed three of these five regions (Regions 1, 6 and 7) as authentic excision hotspots, with a fourth (Region 2) recovered in two of the three replicates, demonstrating that computational prediction identifies biologically active temperate phages in *D. vulgaris* while not being sufficient on its own to establish activity. Similar genome-mining approaches have successfully identified functional prophages in diverse bacterial species [23,25,39]. The concordance observed between the bioinformatic and sequencing datasets supports the use of integrated computational pipelines as reliable tools for identifying candidate prophages suitable for downstream engineering.

The comparative analysis also illustrates an important limitation of purely computational prophage classification. Although PHASTEST assigned Region 5 to the questionable category, this locus consistently generated viral genomes following UV induction, whereas Region 3, classified as intact, showed little or no detectable induction. These observations demonstrate that structural completeness does not necessarily predict biological activity. Prophage induction depends not only on preservation of structural genes but also on regulatory networks controlling excision, replication, and SOS-mediated activation [18,19,44]. Consequently, experimental validation remains an essential complement to computational annotation, particularly when selecting candidate prophages for synthetic biology applications.

The synthetic reconstruction of Region 1 represents an important technical advance of this study. Large bacteriophage genomes remain challenging to manipulate because of their size and complex modular organization [11,28]. By combining PCR, seamless isothermal DNA assembly, and Nanopore sequencing, we successfully reconstructed multiple synthetic prophage genomes with progressively reduced sizes while maintaining complete sequence fidelity across all assembly junctions. Long-read sequencing provided direct confirmation that the assembled genomes precisely matched the intended designs without detectable rearrangements or unintended mutations [41,42,48]. This workflow demonstrates that large endogenous prophage genomes from anaerobic bacteria can be reconstructed reproducibly and provides a practical platform for future genome-scale engineering of temperate phages.

Systematic deletion of prophage lysogeny-associated genomic regions also provides an initial framework for defining the minimal genetic requirements necessary for phage production. Rather than reconstructing only a wild-type genome, we generated a series of progressively truncated variants that preserve structural, replication, and packaging modules while removing selected regions predicted to participate in lysogenic maintenance. Lysogenic control region contains an integrase and a predicted transcriptional regulator together with fifteen open reading frames of unknown function (Table 3); the variants were therefore designed as a length gradient rather than as a set of single-gene deletions. Similar reductionist strategies have been widely used in bacteriophage engineering to define essential genetic modules and construct designer phages with improved properties [26,28,49,50]. Although the present study was not designed to identify the function of individual deleted genes, these constructs establish an experimental platform for future genotype–phenotype analyses aimed at defining essential and dispensable components of temperate phage genomes.

Introduction of the synthetic genomes into *D. vulgaris* followed by UV induction produced reproducible biological responses but did not generate classical lytic plaques. Instead, all three engineered constructs consistently produced localized turbid zones accompanied by accumulation of refractile extracellular material. Several explanations may account for this phenotype. First, the engineered genomes may undergo limited replication but fail to complete efficient virion assembly or host lysis. Second, deletion of lysogeny-associated regions may alter the temporal regulation of phage development, producing incomplete infection cycles. Alternatively, prophage induction may trigger physiological responses within the bacterial population, including altered metabolism, stress responses, extracellular polymer production, or localized biofilm formation. Similar noncanonical phenotypes have been reported for temperate phages and partially functional synthetic phage systems [18,20,27,29]. Because plaque morphology alone cannot distinguish among these possibilities, additional analyses—including transmission electron microscopy, quantitative particle enumeration, viral genome copy number measurements, RNA sequencing, and proteomic characterization—will be required to determine the extent of productive phage replication. The absence of robust lytic activity suggests that essential genes required for productive phage replication and host lysis may reside within other inducible prophage regions, including Regions 2, 5, 6 and 7. Future studies should investigate the biological functions and interactions of these regions to determine their contributions to complete phage activity. In addition, these inducible prophages may serve as attractive candidates for the development of phage-based biosensors and other engineered phage technologies.

The transcriptional analysis further supports the conclusion that UV irradiation successfully activates endogenous prophage gene expression in *D. vulgaris*. Representative structural and lytic genes exhibited coordinated upregulation by 21 h after induction, consistent with activation of the late stages of the temperate phage developmental program [18,20,44]. These data identify 21 h as a practical harvest time for future engineering studies and provide a temporal framework for optimizing phage genome recovery and characterization. Because the constructs reproduce the native Region 1 sequence, transcripts arising from the constructs cannot be distinguished from those of the resident prophage using primers directed at Region 1 genes. The engineered deletion junctions provide construct-specific templates: each junction is contiguous only in the construct, and a junction-spanning primer pair therefore reports on the construct alone. Quantifying junction-spanning transcripts and construct copy number in transformed and mock-transformed cells after UV treatment would establish directly whether the constructs are transcribed and maintained, and is the immediate next step.

Beyond the immediate findings, this study establishes a generalizable pipeline for engineering endogenous prophages directly from bacterial chromosomes. Most synthetic bacteriophage engineering efforts have focused on well-characterized model systems such as T7, λ, or mycobacteriophages [26,27,28,30,31]. In contrast, synthetic manipulation of prophages from environmentally important anaerobic microorganisms remains largely unexplored. Our workflow integrates genome mining, experimental validation, synthetic genome reconstruction, and functional evaluation into a unified platform that should be readily adaptable to other bacteria harboring endogenous prophages. Such approaches may ultimately facilitate the development of customized phages for biotechnology, microbiome engineering, and targeted control of environmentally important bacterial populations [11,14,51].

Several limitations should be acknowledged. Only UV irradiation was used as the inducing stimulus, and therefore prophages responsive to alternative environmental signals, such as mitomycin C, oxidative stress, nutrient limitation, or other DNA-damaging conditions, may not have been detected [19,52,53]. Likewise, the biological activity of the engineered genomes was assessed primarily by spot assays and transcriptional analyses, whereas direct visualization of phage particles, quantitative measurements of burst size, adsorption efficiency, infectivity, and genome replication remain to be determined [10,36]. Finally, only one prophage region was selected for synthetic reconstruction, and future studies should extend this approach to additional inducible prophages identified in the *D. vulgaris* genome. Such comparative analyses will provide a broader understanding of prophage diversity, regulation, and engineering potential within sulfate-reducing bacteria [18,28].

## 5. Conclusions

In conclusion, this study demonstrates that endogenous prophages within *D. vulgaris* can be systematically identified, experimentally validated, synthetically reconstructed, and functionally evaluated using an integrated computational and experimental framework. The close correspondence between bioinformatic prediction and empirical induction validates the use of genome-wide prophage mining for identifying active temperate phages, while the successful assembly of sequence-verified synthetic genomes establishes a practical foundation for large-scale phage engineering in SRB. Although the engineered constructs did not produce canonical lytic plaques under the conditions tested, they elicited reproducible biological responses and provide an important starting point for dissecting the genetic requirements governing temperate phage activation. More broadly, this work expands the synthetic biology toolbox available for anaerobic microorganisms and provides a foundation for future development of programmable phage platforms for microbial engineering, biotechnology, and environmental applications.

## Figures and Tables

**Table 1:** Quantitative profile and structural classification of the seven prophage regions identified in the *Desulfovibrio vulgaris* Hildenborough chromosome by PHASTEST. For each region, the table reports the genomic length (kb), PHASTEST completeness classification (intact, questionable, or incomplete), prediction confidence score, total number of predicted proteins, chromosomal position (bp), and GC content (%). Regions 1, 2, 3, 6, and 7 were classified as intact, Region 5 as questionable, and Region 4 as incomplete, reflecting differences in coding density, gene completeness, and phage-associated marker content. Cell shading indicates the PHASTEST completeness classification: green, intact (score > 90); yellow, questionable (score 70–90); red, incomplete (score < 70).

Region	Region Length (kb)	Completeness	Score	Total Proteins	Region Position (bp)	GC Content (%)
1	37.3	intact	150	46	233,143–270,532	63.63
2	43.6	intact	150	49	1,197,954–1,241,586	64.44
3	43.1	intact	140	40	1,556,471–1,599,625	58.41
4	37.3	incomplete	60	15	1,783,411–1,820,727	59.64
5	24.9	questionable	90	25	2,250,828–2,275,748	62.74
6	33.0	intact	150	46	2,801,325–2,834,336	65.56
7	41.4	intact	150	51	2,936,186–2,977,658	62.54

**Table 2:** Quantitative alignment summary cross-referencing empirically induced *Desulfovibrio vulgaris* phage sequences (IPh_S1–IPh_S3) with computationally predicted prophage regions. For each induced phage replicate, the table lists the most highly aligned genomic coordinates (kb), the corresponding aligned region length (kb), and the matching PHASTEST-predicted prophage region with its full predicted length (kb). Consistent alignment was observed for Regions 1, 5, 6 and 7 across all three replicates (Region 5 was computed as questionable in completeness score by PHASTEST), less frequent alignment was observed for Region 2, whereas Regions 3 and 4 showed no detectable matching sequence under the UV induction conditions used in this study. Text colours identify the prophage region: red, Region 1; purple, Region 2; green, Region 5; blue, Region 6; orange, Region 7.

Sample Name	Most Aligned Region (kb)	Aligned Length (kb)	Corresponding Predicted Region (kb)	Predicted Length (kb)
**IPh_S1**	**233–270**	**37**	**Region 1 (233–270)**	**37**
	**1213–1239**	**26**	**Region 2 (1198–1242)**	**44**
	**2266–2283**	**17**	**Region 5 (2251–2276)**	**25**
	**2799–2834**	**35**	**Region 6 (2801–2834)**	**33**
	**2936–2974**	**38**	**Region 7 (2936–2977)**	**41**
**IPh_S2**	**233–270**	**37**	**Region 1 (233–270)**	**37**
	**1213–1239**	**26**	**Region 2 (1198–1242)**	**44**
	**2256–2294**	**38**	**Region 5 (2251–2276)**	**25**
	**2799–2834**	**35**	**Region 6 (2801–2834)**	**33**
	**2936–2978**	**42**	**Region 7 (2936–2977)**	**41**
**IPh_S3**	**233–271**	**38**	**Region 1 (233–270)**	**37**
	**2262–2294**	**32**	**Region 5 (2251–2276)**	**25**
	**2799–2834**	**35**	**Region 6 (2801–2834)**	**33**
	**2936–2970**	**34**	**Region 7 (2936–2977)**	**41**

**Table 3:** Open reading frames within the lysogeny control region of the Region 1 prophage and their status in each synthetic construct. The two functionally annotated genes (PP_00214 and PP_00230) are shown in bold on a shaded background.

PHASTEST ID	Coordinates (bp)	Strand	Length	PHASTEST Annotation	C1	C2	C3
**PP_00214**	**261,875–262,270**	**+**	**396**	**CopG family transcriptional regulator**	**—**	**—**	***10 bp left***
PP_00215	262,361–262,606	−	246	hypothetical	—	—	✓
PP_00216	262,657–263,778	−	1122	hypothetical	—	—	✓
PP_00217	263,799–264,329	−	531	hypothetical	—	—	✓
PP_00218	264,414–264,803	−	390	hypothetical	—	—	✓
PP_00219	264,855–265,049	+	195	hypothetical	—	—	✓
PP_00220	265,193–265,480	+	288	hypothetical	—	—	✓
PP_00221	265,593–265,769	+	177	hypothetical	—	*trunc*	✓
PP_00222	265,802–266,266	+	465	hypothetical	—	✓	✓
PP_00223	266,347–266,571	+	225	hypothetical	—	✓	✓
PP_00224	266,561–266,812	+	252	hypothetical	—	✓	✓
PP_00225	266,955–267,194	+	240	hypothetical	—	✓	✓
PP_00226	267,203–267,985	+	783	hypothetical	—	✓	✓
PP_00227	267,982–268,305	+	324	hypothetical	—	✓	✓
PP_00228	268,308–268,871	+	564	hypothetical	—	✓	✓
PP_00229	268,861–269,286	+	426	hypothetical	*trunc*	✓	✓
**PP_00230**	**269,283–270,425**	**−**	**1143**	**Integrase (PHAGE_Shigel_Stx_NC_029120)**	**✓**	**✓**	**✓**

✓, deleted; —, retained; trunc, partially removed. Entries in red indicate open reading frames that are only partially removed in the construct concerned.

## Data Availability

The data presented in this study are available in the article. Additional raw data (e.g., sequencing reads) are available from the corresponding author upon reasonable request.

## Supplementary Materials

The following supporting information can be downloaded at: https://www.mdpi.com/article/10.3390/v18101159/s1, Figure S1: RefFinder stability analysis of the four candidate reference genes; Table S1: Sequencing depth across the PHASTEST-predicted prophage regions in the three induced-phage preparations.
