# Supplementary material for: Synthetic Reconstruction and Characterization of Endogenous Bacteriophage Genomes from Desulfovibrio vulgaris
Source: Viruses. 2026 Oct 19;18(10):1159. doi: 10.3390/v18101159 (PMC13709724; doi:10.3390/v18101159)
Supplement: Supplementary file 1 [file viruses-18-01159-s001.zip › viruses-4564341-supplementary.pdf]

Region	Position (kb)	IPh_S1	IPh_S2	IPh_S3	Detected
1	233–271	11.7	61.5	3.2	**3 / 3**
2	1,198–1,242	2.9	9.9	0.1	**2 / 3**
3	1,556–1,600	0.0	0.0	0.0	**0 / 3**
4	1,783–1,821	0.0	0.8	0.0	**0 / 3**
5	2,251–2,276	3.1	22.6	18.3	**3 / 3**
6	2,801–2,834	1.0	9.0	8.1	**3 / 3**
7	2,936–2,978	13.8	27.6	3.3	**3 / 3**

Whole-library statistics

	IPh_S1	IPh_S2	IPh_S3
Mapped reads	441	1,568	593
Mapped bases	1.75 Mb	5.72 Mb	1.70 Mb
Read N50 (bp)	8,220	8,646	6,263
Mean depth, whole chromosome (x)	0.40	1.51	0.42
Background depth outside the seven regions (x)	0.06	0.19	0.16

Table S1. Sequencing depth across the PHASTEST-predicted prophage regions in the three induced-phage preparations. Values are mean read depth (x) across each region. Reads were aligned to the Desulfovibrio vulgaris Hildenborough chromosome (NC_002937.3) with minimap2 (map-ont preset). Depth was calculated from primary and supplementary alignments; secondary alignments were excluded. A region was scored as detected where its mean depth exceeded the background depth outside all seven predicted regions by fivefold or more. The three libraries were barcoded and sequenced on a single flow cell. Because purified phage DNA was amplified by rolling-circle amplification before library preparation, and this amplifies circular templates preferentially, depth indicates detection rather than the abundance of each genome in the original preparation; the values therefore distinguish detection from non-detection but do not support quantitative comparison of induction efficiency between loci.

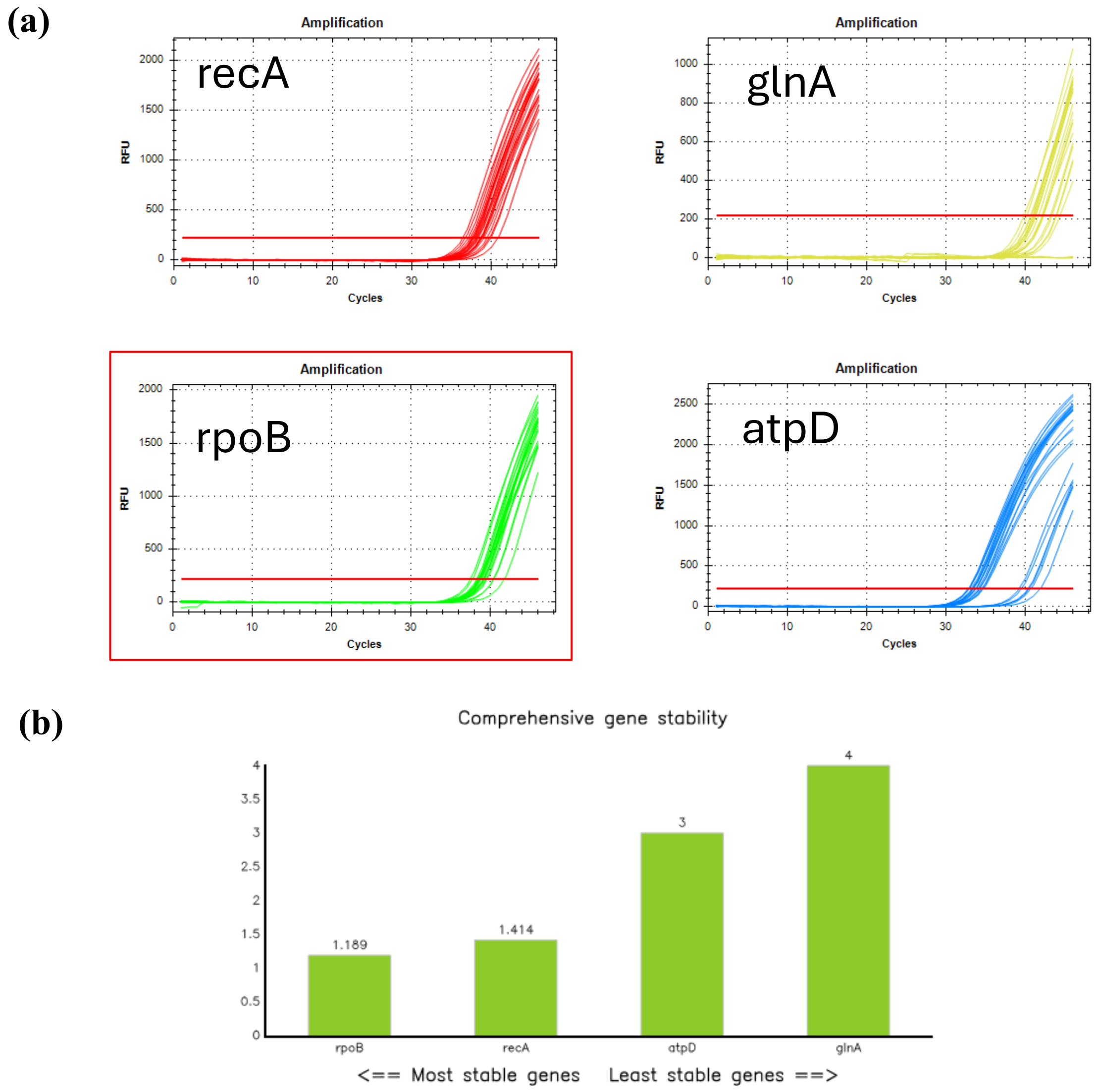

Figure S1. RefFinder stability analysis. (a) amplification curve for each reference gene. (b) RefFinder analysis. Less comprehensive gene stability value represent higher stability of expression throughout samples.
